# 1-year radiological, functional and quality-of-life outcomes in patients with SARS-CoV-2 pneumonia - A prospective observational study

**DOI:** 10.1038/s41533-022-00273-z

**Published:** 2022-03-03

**Authors:** Marco Marando, Tanja Fusi-Schmidhauser, Adriana Tamburello, Lorenzo Grazioli Gauthier, Elia Rigamonti, Gianluca Argentieri, Carla Puligheddu, Alberto Pagnamenta, Antonio Valenti, Marco Pons, Pietro Gianella

**Affiliations:** 1 Department of Internal Medicine, Ospedale Regionale di Lugano, Ente Ospedaliero Cantonale, Lugano, Switzerland; 2 IIMSI - Radiology Department, Ospedale Regionale di Lugano, Ente Ospedaliero Cantonale, Lugano, Switzerland; 3 Department of intensive care, Ospedale Regionale di Mendrisio, Ente Ospedaliero Cantonale, Mendrisio, Switzerland; 4 Clinical Trial Unit, Ente Ospedaliero Cantonale, Lugano, Switzerland; 5 Division of Pneumology, Ospedale Regionale di Lugano, Ente Ospedaliero Cantonale, Lugano, Switzerland; 6 Division of Pneumology, University of Geneva, Geneva, Switzerland

**Keywords:** SARS-CoV-2, Prognosis

## Abstract

All over the world, SARS-CoV-2 pneumonia is causing a significant short and medium-term morbidity and mortality, with reported persisting symptoms, radiological and lung alterations up to 6 months after symptoms onset. Nevertheless, the 1-year impact on affected patients is still poorly known. In this prospective observational study, 39 patients with SARS-CoV-2 pneumonia were recruited from a single COVID-19 hospital in Southern Switzerland. They underwent a 3-month and 1-year follow-ups. At 1 year, 38 patients underwent functional follow-up through lung function tests and six minutes walking test and submitted SF-12 and SGRQ questionnaires about health-related quality of life. At 1 year most of the patients showed a persistence of the radiological and functional abnormalities and a reduction of the health-related quality of life. Thirty patients (96.8%) still presented some residual abnormalities on CT scans (31 patients at 3 months), though with a general reduction of the lesional load in all lung lobes. Twenty patients (52.6%) had persisting lung function tests impairment, with an overall improvement of DLCO. As concerning the functional status, lowest SpO2 during 6MWT increased significantly. Finally, 19 patients (50%) reported a pathological St. George’s Respiratory Questionnaire, and respectively 12 (31.6%) and 11 (28.9%) patients a pathological Short Form Survey-12 in physical and mental components. At 1-year follow-up SARS-CoV-2 pneumonia survivors still present a substantial impairment in radiological and functional findings and in health-related quality of life, despite showing a progressive recovery.

## Introduction

The severe acute respiratory syndrome-coronavirus-2 (SARS-CoV-2) was first diagnosed in December 2019 in Wuhan, China, and on March 11, 2020 has been declared a pandemic by the World Health Organization. SARS-CoV-2 infection is associated with considerable short-term morbidity and mortality^1^. During the acute phase, severe cases typically have lung involvement^2,3^. The mid-term effects of SARS-CoV-2 pneumonia have been elucidated in recent publications, which flag the persistence of alterations in lung function tests and in chest imaging up to 6 months^4–6^ after symptoms onset and the reduction of health-related quality of life^7^. Interestingly, similar observations were made in SARS-CoV^8^ and H7N9^9^ survivors. More recently, Wu et al. reported the results of a 1-year prospective trial of patients hospitalised for severe COVID-19 in China: despite most patients improved dyspnea scores and exercise capacity over time, in a subgroup of patients there was the evidence of persistent physiological and radiographic changes^10^. However, the long-term radiological and functional outcomes and the impact on quality of life in SARS-CoV-2 survivors are still not completely known. Previously, 3-month follow-up results of our cohort were reported:^11^ among SARS-CoV-2 pneumonia survivors, we reported significant radiological and lung function tests abnormalities and an overall decreased quality of life. We therefore conducted a prospective observational study with 1-year follow up aiming to describe radiological and lung function parameters and self-reported health-related quality of life (HRQoL) of SARS-CoV-2 pneumonia survivors.

## Methods

### Case definition

Study participants were diagnosed on the result of a positive real-time reverse-transcriptase polymerase chain reaction (rRT-PCR) assay for SARS-CoV-2.

### Participants and study design

**Fig. 1:**
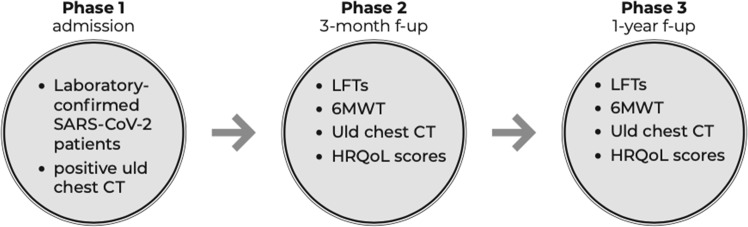
Study timeline. We represent the study timeline, with the 3 phases (admission, 3-month f-up and 1-year f-up) and the exams performed in each phase.

In this prospective observational single-center study we enrolled 39 consecutive laboratory-confirmed COVID-19 patients with pathological findings on a chest ultra-low dose (uld) CT scan performed at hospital admission between March 1 and April 15, 2020. A written informed consent was obtained from all participants. Exclusion criteria were age <18 years, pregnancy and absence of a written informed consent. For all included patients demographic, clinical and laboratory data were collected. Prior to hospital discharge a follow-up visit was planned at 3 months and 1 year after the admission. Patients severity at hospital admission was evaluated by the ISARIC4C score, in both mortality^12^ and deterioration^13^ components. ISARIC4C score is a prospectively validated model, in which higher scores indicate an higher risk of the examined outcome (respectively death and deterioration). At follow-up all patients underwent lung function tests (LFTs), 6-Minute Walk Test (6MWT), an uld chest CT scan and self-reported HRQoL questionnaires (St. George’s Respiratory Questionnaire [SGRQ] and Short Form Survey-12 [SF-12]) (Fig. 1). As stated above, 3-month data of our cohort have been presented in a recent publication^11^. The study was approved by the local ethics committee of Southern Switzerland (2020-01270 CE 3649). A written informed consent was obtained from all the patients. The study complies to the Declaration of Helsinki requirements.

### Chest CT protocol

**Fig. 2:**
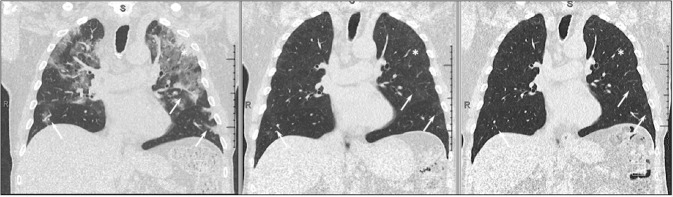
Typical SARS-CoV-2 CT scan lesions and their evolution at 3 and 12 months. Asterisks show complete healing of the areas of consolidation at 3 months. White arrows demonstrate a slower but almost complete resolution of the areas of ground glass opacities at 3 and 12 months.

Uld CT has proven to be more sensitive for the detection of COVID-19 lesions than chest X-ray (CXR)^14^ and international guidelines have also made recommendations in favour of CT for the diagnostic work-up of COVID-19^15^. In addition, experts highlighted the issue of exposition to radiation doses and encouraged the use of low-dose CT scans^16^. In our cohort all patients underwent uld chest CT in supine position at full inspiration, without intravenous contrast medium, using two multi-detector scanners: Siemens Somatom Definition Flash and Siemens Somatom Definition Edge (Siemens, Erlangen, Germany). Scan parameters for uld CT were optimized for a patient with a normal body mass index (BMI between 18.5 and 24.9 kg/m^2^) and with an effective dose varying from 0.14 to 0.5 mSv as reported in the current literature^17,18^. Image analysis and final scores were performed by consensus by two radiologists (G.A., and C.P., with 15 and 20 years of experience in thoracic radiology, respectively) who scored independently and blinded to clinical data. Images were reviewed on a professional picture archiving and communication system (PACS) PC workstation (Philips Intellispace PACS). A semiquantitative scoring system based on the method proposed by Pan et al.^19^ was used to estimate the global pulmonary involvement of all abnormalities on the basis of the area involved. For each lobe the presence of a predominant pattern for ground-glass opacity (GGO), consolidation, fibrosis or parenchymal bands was determined and each of the five lung lobes was visually scored on a scale of 0 to 5, with 0 indicating no involvement; 1, less than 5% involvement; 2, 5–25% involvement; 3, 26–49% involvement; 4, 50–75% involvement; and 5, more than 75% involvement. The total CT score was the sum of the individual lobar scores and ranged from 0 (no involvement) to 25 (maximum involvement). Presence of a pleural effusion, thoracic lymphadenopathy (defined as lymph node size of 10 mm in short-axis dimension) or underlying lung disease such as emphysema or fibrosis were noted but not score. In Fig. 2 we present CT scan lesions of a typical SARS-CoV-2-related pneumonia and the evolution at 3 and 12-month.

### LFTs and QoL assessment

LFTs were conducted in the Division of Pneumology at the Regional Hospital of Lugano, Switzerland, using the Vyntus BODY Plethysmograph (Vyaire Medical, IL, USA) according to the European Respiratory Society (ERS) guidelines^20,21^. We measured both static and dynamic volumes, other than performing bronchodilation tests and assessing diffusing lung capacity for carbon monoxide (DLCO). Since interstitial lung disease and pulmonary vascular diseases are considered the most important lung complications of COVID-19, we defined as abnormal LFT the presence of a DLCO < lower limit of normal (LLN) and/or of a TLC < LLN. Thereafter, patients underwent a 6MWT and self-reported QoL questionnaires (SGRQ and SF-12) were submitted by all participants^22,23^. Pathological SGRQ and SF-12 scores, in their respective components, were defined by a higher score than that reported in literature and validated in the general population (normal references range values indicated in Table 5). 6MWT results were described as distance walked in metres and in % of predicted for healthy individuals of the same age, sex, height and weight and as lowest SpO2 during 6MWT. As concerning QoL questionnaires, while the SGRQ is widely used to evaluate patients with respiratory diseases, SF-12, in its physical and mental components, provides a multidimensional assessment of patients, especially with regard to their role limitations as a result of emotional problems, mental health, physical pain, and general health perception.

### Statistical analysis

Quantitative data are reported as mean ± standard deviation (SD) or as median with the 25^th^ and 75^th^ percentile, unless otherwise indicated, whereas qualitative data are summarized as absolute values with the corresponding percentages. Parametric or non-parametric paired tests were used to compare two time-points estimates (paired Student *t*-test, or Wilcoxon-rank test). A repeated-measures analysis of variance (ANOVA) was used to compare variables assessed at three different time-points after checking for normality and homoscedasticity with the conventional tests. By violation of these assumptions the non-parametric repeated ANOVA (Friedman test) was used. When the *F*-ratio of the ANOVA or the Friedman test reached a critical level (corresponding to a *P* < 0.05) post hoc analysis with *P*-value adjustment for multiple comparison was used. Categorical paired nominal data at two time-points were compared with the McNemar test. By three time-points *P*-values were adjusted for multiple comparisons. All tests were performed two-sided and a *P*-value < 0.05 was considered statistical significant. Statistical analysis was performed using Stata Version 15 (StatCorp.LP, College Station, TX, USA).

### Reporting summary

Further information on research design is available in the Nature Research Reporting Summary linked to this article.

## Results

### Participants

An overview of participants’ main demographic and clinical characteristics is shown in Table 1. Patient severity as evaluated by the ISARIC4C score, was 6.1 ± 2.9 and 421.6 ± 73.8 in respectively mortality and deterioration components. Notably, at 1 year there was just one dropout, thus the study sample was 38.

**Table 1:** Clinical characteristics of included patients.

	Normal range	Overall – 1 year (*n* = 38)	CT improvement or normalised at 1 year (*n* = 32)	CT not improving at 1 year (*n* = 6)	*P-*value
Age (years, median and IQR)		64.5 (52.7–72.2)	65.5 (55–72.75)	53.5 (49.5 –71.25)	0.28
Sex (female, n and %)		8 (21)	24 (75)	0 (0)	0.001
BMI > 25 kg/m^2^ (*n* and %)		30 (78.9)	20 (62.5)	6 (100)	0.15
Active smokers (*n* and %)		3 (7.9)	3 (9.4)	0 (0)	1
Previous smokers (*n* and %)		12 (30.8)	11 (34.4)	1 (16.7)	0.64
Length of stay (days, median and IQR)		15 (12–22.5)	14.5 (12–20.75)	22 (7.2–39.5)	0.49
Hypertension (*n* and %)		14 (36.8)	13 (40.6)	1 (16.7)	0.38
Diabetes (*n* and %)		5 (13.1)	5 (15.6)	0 (0)	0.57
Cardiovascular diseases (*n* and %)		7 (18.4)	6 (18.7)	1 (16.7)	1
Coronary heart disease (*n* and %)		4 (10.5)	3 (9.4)	1 (16.7)	0.51
Chronic respiratory diseases (*n* and %)		8 (21)	8 (25)	0 (0)	0.32
COPD (*n* and %)		3 (7.9)	3 (9.4)	0 (0)	1
Asthma (*n* and %)		5 (13.1)	5 (15.6)	0 (0)	0.57
Chronic kidney disease (*n* and %)		3 (7.9)	2 (6.2)	1 (16.7)	0.41
Malignancy (*n* and %)		4 (10.5)	4 (12.5)	0 (0)	1
Depression (*n* and %)		4 (10.5)	3 (9.4)	1 (16.7)	0.51
Intensive care unit admission (*n* and %)		10 (26.3)	7 (21.9)	3 (50)	0.31
Invasive mechanical ventilation (*n* and %)		7 (18.4)	5 (15.6)	2 (33.3)	0.61
Rehabilitation after discharge (*n* and %)		7 (18.4)	5 (15.6)	2 (33.3)	0.61
ISARIC4C score – mortality on admission (mean ± SD)		6.1 ± 2.9	6.3 ± 2.8	4.8 ± 3.5	0.26
ISARIC4C score – deterioration on admission (mean ± SD)		421.6 ± 73.8	429.5 ± 65	379.3 ± 107.7	0.13
Peak PCR on admission (mg/l) (mean ± SD)	1–5	185.7 ± 147.4	179.3 ± 142.3	230.8 ± 189.5	0.44
Peak LDH on admission (U/l) (mean ± SD)	< 500	653.2 ± 348.5	644.6 ± 351.4	690.5 ± 394.1	0.77
Peak leukocytes on admission (G/l) (mean ± SD)	4.2–10	8.9 ± 4.8	9 ± 4.6	8.7 ± 4.4	0.90
Peak lymphopenia on admission (G/l) (mean ± SD)	1.5–2.5	0.7 ± 0.2	0.7 ± 0.2	0.6 ± 1.2	0.53
Peak thrombopenia on admission (G/l) (mean ± SD)	150–400	185.5 ± 81.9	191.6 ± 87.9	149 ± 33.6	0.25
Peak d-dimer on admission (mg/l) (mean ± SD)	< 0.5	4.8 ± 10	3.7 ± 6.3	9.8 ± 20.4	0.19
Lymphocytes on admission (G/l) (mean ± SD)	1.5–2.5	0.8 ± 0.3	0.8 ± 0.3	0.9 ± 0.4	0.61
Leukocytes on admission (G/l) (mean ± SD)	4.2–10	5.5 ± 2.3	5.8 ± 2.4	4.2 ± 1	0.14
Thrombocytes on admission (G/l) (mean ± SD)	150–400	189.9 ± 74.7	195.1 ± 79	156.7 ± 45.1	0.26
PaO2 on admission (kPa) (mean ± SD)	> 8	9.3 ± 1.4	9.2 ± 1.2	9.9 ± 2.1	0.27
nt-proBNP on admission (ng/l) (mean ± SD)	< 450	275.7 ± 253.7	277.9 ± 225.6	261 ± 453.5	0.91
D-dimer on admission (mg/l) (mean ± SD)	< 0.5	1.1 ± 0.8	1.2 ± 0.9	0.9 ± 0.8	0.43
Antibiotics (*n* and %)		24 (61.5)	20 (62.5)	3 (50)	0.66
Hydroxychloroquine (*n* and %)		32 (82)	27 (84.4)	4 (66.7)	0.30
Remdesevir (*n* and %)		2 (5.1)	1 (3.1)	0 (0)	1
Tocilizumab (*n* and %)		4 (10.2)	3 (9.4)	0 (0)	1
Lopinavir-Ritonavir (*n* and %)		21 (53.8)	15 (46.9)	5 (83.3)	0.18
ACE-I, ARB treatment (*n* and %)		11 (28.2)	10 (31.3)	1 (16.7)	0.65
Corticosteroids during hospitalisation (*n* and %)		0 (0)	0 (0)	0 (0)	-
Oxygen therapy during hospitalisation (*n* and %)		36 (94.7)	31 (96.9)	5 (83.3)	0.29
Immunosuppressive therapy on admission* (*n* and %)		1 (2.6)	1 (3.1)	0 (0)	1
Anticoagulation on admission (*n* and %)		4 (10.2)	4 (12.5)	0 (0)	1
Antiplatelet therapy on admission (*n* and %)		7 (17.9)	6 (18.7)	1 (16.7)	1

*Immunosuppressive therapy on admission was defined as the habitual intake of more than 20 mg/day of prednisone or of an equivalent dose of other corticosteroids, and/or of calcineurin inhibitors, and/or of antiproliferative agents, and/or of mTOR inhibitors and/or of any treatment that interferes with the physiological immune response.

### Analysis of the radiological findings

Thirty-one patients (81.6%) showed a pathological CT scan at three-month follow-up, therefore undergoing chest CT also at 12 months. In our cohort, 32 (84.2%) patients showed an improved or normal CT scan at 1 year time-point (defined as both improving CT scan at 1-year or normalized CT scan at 3-month follow-up), while 6 (15.8%) patients had a stable or worsening CT scan. Nevertheless, at 1 year almost every patient with pathological findings on a CT scan at 3 months had some residual radiological abnormality. According to the CT score, every lung lobe and the overall pulmonary involvement showed a CT score reduction at 3- and 12-month follow-ups (Tables 2 and 3). The most frequently reported abnormal finding was the presence of fibrous bands, followed by GGO and consolidations.

**Table 2:** Radiological characteristics on admission, at three-month and 1-year follow-ups.

	CT on admission (*n* = 39)	CT at 3 months (*n* = 39)	CT at 12 months (*n* = 31)
Ground glass opacities (*n* and %)	34 (87.1)	23 (59)	21 (67.7)
Consolidations (*n* and %)	17 (43.6)	1 (2.6)*	3 (9.7)*
Fibrous bands (*n* and %)	28 (71.8)	27 (69.2)	23 (74.2)
Pathological CT scans (n and %)	39 (100)	32 (82)	30 (96.8)

*P-value < 0.05 as compared with on admission.

**Table 3:** CT score (0–5) per lobe and overall (0–25) on admission, at three-month and at 1-year follow-ups.

	CT on admission (*n* = 39)	CT at 3 months (*n* = 39)	CT at 12 months (*n* = 31)
Right upper lobe (mean ± SD)	2.5 ± 1.2	1.4 ± 1.2^#^	1.2 ± 1.1^#§^
Middle lobe (mean ± SD)	2 ± 1.3	1.2 ± 1.1^#^	0.7 ± 0.6^#§^
Right lower lobe (mean ± SD)	2.7 ± 1.1	1.5 ± 1.2*	1.3 ± 1.1^#§^
Left upper lobe (mean ± SD)	2.4 ± 1.4	1.4 ± 1.3*	1.1 ± 1^#§^
Left lower lobe (mean ± SD)	2.7 ± 1	1.5 ± 1.3^#^	1.3 ± 1^#§^
CT score overall (mean ± SD)	12.9 ± 4.5	8.6 ± 4.3^#^	5.5 ± 3.9^#§^

*P-value < 0.05 as compared with on admission.

^#^*P*-value < 0.001 as compared with on admission.

^§^*P*-value < 0.05 as compared with 3 months.

### Lung function test and quality of life tests analysis

**Fig. 3:**
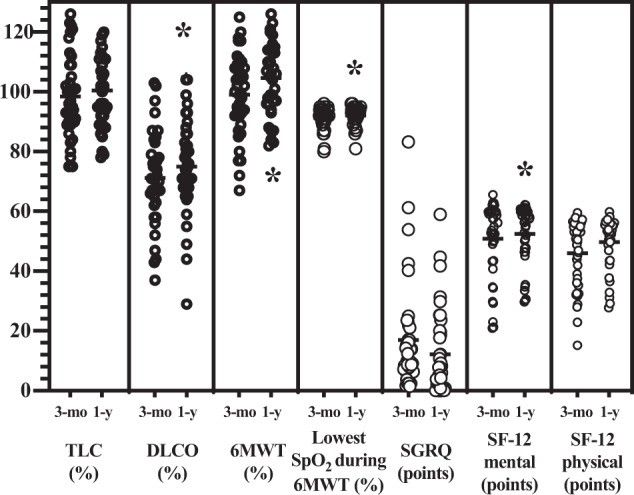
Comparison of lung function tests, 6 min walk test, lowest SpO2 during 6MWT and health-related quality of life questionnaires (SGRQ and SF-12) at 3- and 12-month follow-ups. Each variable was represented as mean ± SD or median and 25^th^–75^th^ percentile as appropriate.

Lung function tests abnormalities (i.e., reduced DLCO and/or restriction) were found in half of all patients. The most frequent alteration was an abnormal DLCO, while only very few patients had restriction. Nevertheless, at 1 year patients showed an overall improvement in DLCO (*P* = 0.002). Moreover, all patients presented an improving lowest SpO_2_ during 6MWT (*P* < 0.001) (Table 4). Half of the participants had an abnormal SGRQ, while only about one third of all patients reported an abnormal SF-12 score, both in the physical and mental components. At 1 year patients expressed overall less respiratory symptoms as reported in SGRQ (*P* = 0.009): in particular, there was an improvement in impact (*P* = 0.04) and symptoms (*P* = 0.03) domains. Likewise, at 1 year patients reported less physical limitations (SF-12 physical) as compared to the 3-month follow-up (*P* = 0.005). On the opposite, we could not find any difference in the SF-12 mental score (Table 5). In Fig. 3 we represented the comparison of lung function tests, 6-minute walk test, lowest SpO2 during 6MWT and health-related quality of life between 3-12-month follow-ups.

**Table 4:** Lung function tests and functional tests at three-month and 1-year follow-ups.

	Overall at 3 mo (*n* = 39)	Overall at 12 mo (*n* = 38)	*P-*value	CT improvement or normalised at 1 year (*n* = 32)	CT not improving at 1 year (*n* = 6)	*P-*value
FEV 1 (l) (mean ± SD)	2.9 ± 0.7	3 ± 0.8	0.07	3 ± 0.7	3.1 ± 0.9	0.6
FEV 1 (% of predicted) (mean ± SD)	93.4 ± 16.1	96.5 ± 17.9	0.02	97 ± 18	94 ± 17.9	0.71
FVC (l) (mean ± SD)	3.7 ± 0.9	3.9 ± 0.9	0.008	3.9 ± 0.9	3.9 ± 1.3	0.91
FVC (% of predicted) (mean ± SD)	92.5 ± 13.6	96.2 ± 14.6	0.25	97.5 ± 13.5	89.5 ± 19.3	0.22
Obstruction (*n* and %)	3 (7.7)	4 (10.5)	1	4 (12.5)	0 (0)	1
Restriction (*n* and %)	3 (7.7)	2 (5.3)	1	1 (3.1)	1 (16.7)	0.29
TLC (%) (mean ± SD)	98.5 ± 13.6	100.4 ± 14	0.11	101.8 ± 14.5	93.2 ± 9	0.17
Abnormal DLCO (n and %)	22 (56.4)	18 (47.4)	0.50	16 (50)	2 (33.3)	0.66
DLCO (% of predicted) (mean ± SD)	71.3 ± 15.5	75 ± 15.8	0.002	74.9 ± 16.8	76 ± 9.8	0.87
LFTs abnormalities (*n* and %)	25 (64.1)	20 (52.6)	0.36	20 (62.5)	3 (50)	0.66
6MWT (m) (mean ± SD)	539.3 ± 102.8	556.4 ± 92.1	0.09	559.6 ± 90.1	538.2 ± 108.8	0.6
6MWT (% of predicted) (mean ± SD)	99 ± 13.6	104.6 ± 12.7	0.07	105.8 ± 13.3	98.8 ± 7.6	0.23
SpO_2_ at rest (%) (median and 25^th^–75^th^)	96 (95–97)	96 (95–97)	0.17	96 (95–97)	97 (95.5–98)	0.16
Lowest SpO_2_ during 6MWT (%) (median and 25^th^–75^th^)	92 (90–94)	93 (92–94)	<0.001	93 (92–94)	93 (88.5–95.25)	0.86
mMRC score (≥2) (*n* and %)	6 (15.4)	6 (15.8)	0.34	6 (18.7)	0 (0)	0.56

*LFTs* lung function tests.

**Table 5:** Health-related quality of life assessment at three-month and 1-year follow-ups.

	Normal reference range values in general population	Overall at 3 mo (*n* = 39)	Overall at 12 mo (*n* = 38)	*P*-value	CT improvement or normalised at 1 year (*n* = 32)	CT not improving at 1 year (n = 6)	*P-value*
St. George symptoms (median and 25^th^–75^th^)	12 (9–15)	16.3 (10.4–29.8)	13.5 (3.6–20.25)	0.07	13.5 (5.4–19.6)	7 (0–22.6)	0.43
St. George activity (median and 25^th^–75^th^)	9 (7–12)	19 (12.2–41.4)	8.7 (0–41.5)	0.28	14.7 (0–47.2)	3.1 (0–18)	0.32
St. George impact (median and 25^th^–75^th^)	2 (1–3)	4 (0–11.5)	0 (0–5.6)	0.04	0 (0–8.6)	0.95 (0–2.5)	0.62
St. George total (median and 25^th^–75^th^)	6 (5–7)	9.9 (7.7–21)	6.9 (1.1–19.8)	0.07	8.1 (1–22.8)	5.2 (1.5–8.8)	0.38
Abnormal St. George total (*n* and %)	-	31 (79.5)	19 (50)	0.009	15 (46.9)	4 (66.7)	0.66
SF-12 physical (median and 25^th^–75^th^)	>50	50.5 (36.1–55)	53.5 (47–55.5)	0.005	52.8 (43.5–55.4)	55.1 (52.3–57.1)	0.16
SF-12 mental (median and 25^th^–75^th^)	>50	54.9 (43.5–59.8)	57.1 (47.6–59.9)	0.4	56.5 (46.7–60.5)	58.8 (49–60.4)	0.54
Abnormal SF-12 physical (*n* and %)	-	19 (48.7)	12 (31.6)	0.16	11 (34.4)	1 (16.7)	0.64
Abnormal SF-12 mental (*n* and %)	-	12 (30.8)	11 (28.9)	>0.99	10 (31.3)	1 (16.7)	0.65

### Univariate analysis results

In the univariate analysis, only female sex category was associated with radiological improvement at 1 year, and notably there was no association between pulmonary functions and quality of life scores and CT scan improvement.

### Socio-economic analysis

As we did for the 3-month follow-up^11^, we investigated the socio-economic impact of COVID-19: while at 3 months 4 (10.2%) patients declared to have stopped their working activity due to respiratory symptoms, at 1 year only 2 (5.3%) patients had to stop working due to persisting symptoms. The remaining patients (36, 94.7%) declared either they did not develop any restriction in their activity, to be retired, housewife(s), or to have a disability allowance.

## Discussion

In our cohort of patients 1 year after SARS-CoV-2 pneumonia, we revealed persisting substantial alterations in radiological findings, pulmonary function tests and patient-reported health-related questionnaires. Up to 1 year after hospital admission, 96.8% of patients who had an abnormal chest CT at 3-month follow-up had persisting radiological signs of lung injury (mostly fibrous bands and GGO), in spite of showing an improvement in the CT score in all lung lobes. As for the lung functions, abnormalities were found in 52.6% of patients, mostly DLCO reduction, while restriction was diagnosed in only 5.3% patients. As concerning the functional status, the lowest SpO_2_ during 6MWT showed a progressive improvement, with a median value of 93% (92–94). Finally, 50% of patients still reported disturbing respiratory symptoms on SGRQ, whereas SF-12 abnormalities in physical and mental components were reported respectively in 31.6% and 28.9% of patients, with an improvement at 1-year in the SF-12 physical and St. George impact domains.

These results show that the extent of the noxious effects of SARS-CoV-2 pneumonia on survivors continue up to 1 year after the diagnosis, despite showing a progressive slow improvement.

Many authors have highlighted the importance of follow-up of patients affected by SARS-CoV-2 pneumonia^24–28^, in the light of the increasingly number of studies who reported radiological and functional sequelae other than the persistence of disturbing symptoms up to 6 months after the hospital admission, the so-called long COVID. Moreover, similar consequences were described following the past epidemics (such as influenza or SARS)^29,30^, which prompted a particular interest on these aspects. As for SARS-CoV-2 sequelae, Anastasio et al. described a reduction of respiratory functions and exercise capacity secondary to SARS-COV-2 pneumonia at 4 months after hospital admission, mostly in patients who developed ARDS during the acute phase^7^. Tabatabae et al. reported residual radiological disease in about half of patients at 3 months, mostly in the subgroup admitted to an intensive care unit (ICU)^31^. More recently, Guler et al. revealed at 4-month follow-up functional impairments and alterations in physical performance that were more pronounced in patients with previously severe COVID-19 clinical courses, compared to those with mild disease^32^ and Huang et al. reported the persistence of at least one symptom and respiratory function impairment, particularly a reduced DLCO at 6 months^5^.

We also have recently reported the results of a 3-month prospective cohort study:^11^ compared to the 3-month timepoint, even if the percentage of patients with any radiological abnormalities is almost superimposable, at 1 year overall radiological involvement was reduced. As for quality of life scores, at 1 year there was an overall reduction in the number of patients with respiratory symptoms as measured by SGRQ, particularly in the impact component, whereas there was no difference in SF-12 in both physical or mental components as compared to the 3-month follow-up. Finally, although in front of an overall persistent respiratory involvement, there was a significant improvement in FEV1, DLCO and lowest SpO2 during 6MWT. While at 3-month we did not find any predictor of radiological improvement, at 1-year follow-up we found that female sex category was associated with radiological improvement.

Data about 1-year outcome is actually scarce. Wu et al.^10^ reported the results of a 1-year prospective trial of 83 patients hospitalised for severe COVID-19 in China, highlighting persisting physiological and radiographic alterations in almost a third of survivors. Chen et al.^33^ reported significant radiological recovery in CT scans in a small cohort of 41 patients, with a median CT score of 0 point, with respect to a median score at hospital admission of 4 points. Becker et al.^34^ reported that 70% of severe COVID-19 survivors developed symptoms of long COVID. Finally, Huang et al.^35^ recently reported a large longitudinal cohort study involving 1276 participants, showing at least one residual symptom in 49% of patients (mostly dyspnoea), significant alteration in DLCO persisting up to 1 year after hospital discharge (54% in critically ill patients and 20–30% in moderately ill patients) and that 12% of patients did not return to work after 1 year.

Our current study confirms that health consequences of SARS-CoV-2 pneumonia can persist up to 1 year after hospital admission, endorsing the call to a structured follow-up of these patients. It also underpins a relevant socio-economic impact of SARS-CoV-2 on society, as considering the number of patients recovering from SARS-CoV-2 pneumonia all over the world, justifying further research focusing on long-term health and economic consequences of this condition.

Our study has several limitations. This trial is monocentric and the sample is relatively small, limiting the generalizability of our results. Furthermore, every patient in the study cohort was diagnosed with pneumonia and approximately 75% of the included patients were not admitted to an ICU, with an overall ISARIC4C score of 6.1 ± 2.9 and 421.6 ± 73.8 in respectively mortality and deterioration components. For these reasons the validity of our results cannot be reasonably extrapolated to asymptomatic or critically ill SARS-CoV-2 patients. Moreover, in assessing quality of life questionnaires we utilized referenced values previously reported in the literature. However, these ranges were validated in the general population^22,23^. Finally, it should be noted that we included the very first patients diagnosed with SARS-CoV-2 pneumonia in Ticino, Switzerland, and one of the first SARS-CoV-2 confirmed patients in Europe. These patients were treated differently than actually recommended (anti-viral therapies, no corticosteroids), and this could have influenced the presented outcomes.

Nevertheless, this trial has several strengths. The whole cohort was prospectively and systematically assessed with minimal drop-out and it added precious information on 1-year outcome, reporting radiological findings based on ultra-low dose computed tomography, complete respiratory function tests and quality of life questionnaires results. In particular, 3-timepoint thoracic CT scan have been rarely reported in prospective study with a similar follow-up. Specifically, 1-year chest CT follow-up was only performed on 83 patients by Wu et al.^10^, on 128 among 1276 patients by Huang et al.^35^ and on 41 patients by Chen et al.^33^.

In conclusion, at 1 year after hospital admission our cohort of patients affected by SARS-CoV-2 pneumonia had persisting radiological and functional impairment and a significantly decreased quality of life, despite showing a progressive and substantial recovery. These results endorse the call to a structured follow-up and provide information to clinicians caring for survivors of SARS-CoV-2 pneumonia. In light of these results, research of radiological and functional outcomes on a longer follow-up and of the role of rehabilitation and psychological services in the management of such patients is warranted.

## Supplementary information

REPORTING SUMMARY

## Data Availability

The de-identified datasets generated and analyzed during the current study are available from the corresponding author on reasonable request.
