# Supplementary material for: 1-year radiological, functional and quality-of-life outcomes in patients with SARS-CoV-2 pneumonia - A prospective observational study
Source: NPJ Prim Care Respir Med. 2022 Mar 3;32:8. doi: 10.1038/s41533-022-00273-z (PMC8894490; doi:10.1038/s41533-022-00273-z)
Supplement: Supplementary file 1 — REPORTING SUMMARY [file 41533_2022_273_MOESM1_ESM.pdf]

nature portfolio

Corresponding author(s): Dr. Adriana Tamburello

Last updated by author(s): 13.11.2021

Reporting Summary

Nature Portfolio wishes to improve the reproducibility of the work that we publish. This form provides structure for consistency and transparency in reporting. For further information on Nature Portfolio policies, see our Editorial Policies and the Editorial Policy Checklist.

Statistics

For all statistical analyses, confirm that the following items are present in the figure legend, table legend, main text, or Methods section.

n/a	Confirmed	
☐	☒	The exact sample size (n) for each experimental group/condition, given as a discrete number and unit of measurement
☐	☒	A statement on whether measurements were taken from distinct samples or whether the same sample was measured repeatedly
☐	☒	The statistical test(s) used AND whether they are one- or two-sided<br>*Only common tests should be described solely by name; describe more complex techniques in the Methods section.*
☐	☒	A description of all covariates tested
☐	☒	A description of any assumptions or corrections, such as tests of normality and adjustment for multiple comparisons
☐	☒	A full description of the statistical parameters including central tendency (e.g. means) or other basic estimates (e.g. regression coefficient) AND variation (e.g. standard deviation) or associated estimates of uncertainty (e.g. confidence intervals)
☐	☒	For null hypothesis testing, the test statistic (e.g. F, t, r) with confidence intervals, effect sizes, degrees of freedom and P value noted<br>*Give P values as exact values whenever suitable.*
☒	☐	For Bayesian analysis, information on the choice of priors and Markov chain Monte Carlo settings
☒	☐	For hierarchical and complex designs, identification of the appropriate level for tests and full reporting of outcomes
☒	☐	Estimates of effect sizes (e.g. Cohen's d, Pearson's r), indicating how they were calculated

Our web collection on statistics for biologists contains articles on many of the points above.

Software and code

Policy information about availability of computer code

Data collection	Microsoft Excel (MAC, 2019).
Data analysis	Statistical analysis was performed using Stata Version 15 (StatCorp.LP, College Station, TX, USA).

For manuscripts utilizing custom algorithms or software that are central to the research but not yet described in published literature, software must be made available to editors and reviewers. We strongly encourage code deposition in a community repository (e.g. GitHub). See the Nature Portfolio guidelines for submitting code & software for further information.

Data

Policy information about availability of data

All manuscripts must include a data availability statement. This statement should provide the following information, where applicable:

- Accession codes, unique identifiers, or web links for publicly available datasets
- A description of any restrictions on data availability
- For clinical datasets or third party data, please ensure that the statement adheres to our policy

The datasets used and analyzed during the current study are available from the corresponding author on reasonable request.

Field-specific reporting

Please select the one below that is the best fit for your research. If you are not sure, read the appropriate sections before making your selection.

☒ Life sciences ☐ Behavioural & social sciences ☐ Ecological, evolutionary & environmental sciences

For a reference copy of the document with all sections, see nature.com/documents/nr-reporting-summary-flat.pdf

Life sciences study design

All studies must disclose on these points even when the disclosure is negative.

Sample size	We enrolled 39 consecutive laboratory-confirmed COVID-19 patients with pathological findings on a chest ultra-low dose (uld) CT scan performed at hospital admission between March 1 and April 15, 2020,
Data exclusions	No available patients were excluded. Exclusion criteria were age < 18 years, pregnancy and absence of a written informed consent.
Replication	Observational study. The study could be replicated in a comparable population.
Randomization	We conducted an observational study.
Blinding	There was no blinding, having conducted an observational study.

Reporting for specific materials, systems and methods

We require information from authors about some types of materials, experimental systems and methods used in many studies. Here, indicate whether each material, system or method listed is relevant to your study. If you are not sure if a list item applies to your research, read the appropriate section before selecting a response.

Materials & experimental systems

n/a	Involved in the study
☒	☐ Antibodies
☒	☐ Eukaryotic cell lines
☒	☐ Palaeontology and archaeology
☒	☐ Animals and other organisms
☐	☒ Human research participants
☒	☐ Clinical data
☒	☐ Dual use research of concern

Methods

n/a	Involved in the study
☒	☐ ChIP-seq
☒	☐ Flow cytometry
☒	☐ MRI-based neuroimaging

Human research participants

Policy information about studies involving human research participants

Population characteristics	We recruited 39 consecutive SARS-CoV-2 positive patients. Median age was 62.5, 23.1% of total were female.
Recruitment	We enrolled 39 consecutive laboratory-confirmed COVID-19 patients with pathological findings on a chest ultra-low dose (uld) CT scan performed at hospital admission between March 1 and April 15, 2020
Ethics oversight	The study was approved by the local ethics committee of Southern Switzerland (2020-01270 CE 3649).

Note that full information on the approval of the study protocol must also be provided in the manuscript.
